# A scoping review linking early childhood caries to violence, neglect, internally displaced, migrant and refugee status

**DOI:** 10.1186/s12903-023-03459-0

**Published:** 2023-10-11

**Authors:** Morenike Oluwatoyin Folayan, Robert J. Schroth, Imen Ayouni, Arthemon Nguweneza, Arheiam Arheiam, Ola B. Al-Batayneh, Jorma I. Virtanen, Balgis Gaffar, Duangporn Duangthip, Ivy Guofang Sun, Simin Mohebbi, Carlos A. Feldens, Maha El Tantawi

**Affiliations:** 1 Early Childhood Caries Advocacy Group, Winnipeg, Canada; 2 Department of Child Dental Health, Obafemi Awolowo University, Ile-Ife, Nigeria; 3 Dr. Gerald Niznick College of Dentistry, University of Manitoba, Winnipeg, Canada; 4 Department of pediatrics and child health, Faculty of Health Sciences, University of Cape Town, Cape Town, South Africa; 5 Division of Human Genetics, Department of Pathology, Faculty of Health Sciences, University of Cape Town, Cape Town, South Africa; 6 Department of Community and Preventive Dentistry, Faculty of Dentistry, University of Benghazi, Benghazi, Libya; 7 Department of Orthodontics, Pediatric and Community Dentistry, College of Dental Medicine, University of Sharjah, PO Box 27272, Sharjah, United Arab Emirates; 8 Preventive Dentistry Department, Jordan University of Science and Technology, Irbid, Jordan; 9 Faculty of Medicine, University of Bergen, Bergen, Norway; 10 Department of Preventive Dental Sciences, College of Dentistry, Imam Abdulrahman bin Faisal University, Dammam, Saudi Arabia; 11 Faculty of Dentistry, The University of Hong Kong, Hong Kong SAR, China; 12 Community Oral Health Department, School of Dentistry, Tehran University of Medical Sciences, Tehran, Iran; 13 Department of Pediatric Dentistry, Universidade Luterana do Brasil, Canoas, Brazil; 14 Department of Pediatric Dentistry and Dental Public Health, Faculty of Dentistry, Alexandria University, Alexandria, Egypt

**Keywords:** Wars and conflicts, Peace, Internally displaced persons, Early Childhood Caries, Sustainable development goals

## Abstract

**Background:**

The aim of the scoping review was to identify and synthesize the available literature concerning the relationship between the status of refugees, migrants, and internally displaced persons (IDPs) and Early Childhood Caries (ECC) as it relates to the United Nation’s Sustainable Development Goal 16 (SDG 16).

**Methods:**

Data regarding the links between the status of refugees, migrants, and internally displaced persons (IDPs) and Early Childhood Caries (ECC), and the associations between ECC and maternal and child exposure to physical and sexual abuse, insecurity, crime, exploitation, torture, and displacement were extracted. The search was carried out in January 2023 across three databases (PubMed, Web of Science, and Scopus). Only publications in English with accessible full texts were included. Descriptive statistics were utilized to summarize the categories of the retrieved papers, and graphical representation was employed for visualization purposes. The relationships between the publications and each of the 10 targets of Sustainable Development Goal 16 (SDG 16) were also assessed.

**Results:**

Forty-five studies were reviewed. Most studies (42.2%) originated from the Americas Regions, while no studies were identified from the Africa Region. A significant portion (46.7%) of the papers focused on abuse, violence, and neglect as risk factors for ECC. Migrants, refugees, and IDPs were the most investigated populations (44.4%). Only one study specifically focused on IDPs and migrants respectively. The prevalence of untreated caries was higher among migrants, refugees, and IDPs compared to the host community, ECC was more prevalent among children who experienced abuse, neglect, or were in protective care. The was no clear direction on the associations between ECC and intimate partner violence, adverse childhood experiences, and wars. In terms of the SDGs, the reviewed publications addressed four targets (SDG16.1, SDG16.2, SDG16.3, and SDG16.5) out of the ten targets outlined in SDG 16.

**Conclusion:**

There is available evidence regarding the connections between ECC and war, refugees, migration, violence, and neglect, as outlined in SDG 16. Future studies are needed to investigate how forced movements directly affects ECC status, how disruptions of peace and stability is a risk factor for ECC, and the associations between ECC and other indicators related to SDG 16 targets.

**Supplementary Information:**

The online version contains supplementary material available at 10.1186/s12903-023-03459-0.

## Introduction

The oral health of children is influenced by various family and community related factors [1], which are encompassed by the sustainable development goals. Therefore, it is plausible to assume that addressing the United Nations’ sustainable development goals could ultimately have a positive impact on reducing the global burden of Early Childhood Caries (ECC). ECC is defined as the presence of tooth decay in primary teeth among children under the age of 71 months [2]. Sustainable Development Goal (SDG) 16, established by the United Nations, promotes the development of “peaceful and inclusive societies for sustainable development, provides access to justice for all, and builds effective, accountable, and inclusive institutions at all levels” [3]. The SDG 16 has 10 target indicators ranging from 16.1 that aims to reduce violence everywhere to 16 − 10 that ensures public access to information and protocol for fundamental freedoms.

Peace is defined as the absence of civil unrest or disturbance. It represents a condition of safety and organization within a community, upheld by legal or customary means, harmonious personal relationships, and a state or period of mutual agreement between governments [4]. Peace is an integral aspect of public health and closely intertwined with the right to health as fundamental human rights [4–7]. Establishing strong institutions is crucial for upholding peace, maintaining law and order, and effectively mobilizing human, financial, and other resources for the implementation of programs and initiatives that foster peace and promote health, including oral health [8, 9].

When the state of peace is disrupted and the institutions responsible for maintaining law and order are compromised, the well-being of women and children is disproportionately affected [10, 11]. Wars often result in austerity measures that contribute to child poverty, low birth weight, and declining health outcomes [18]. Furthermore, conflicts and wars disrupt the access of women and children to essential oral health care services, which play a crucial role in preserving oral health and preventing oral diseases [10]. Austerity measures commonly lead to food rationing and reduced access to sugar [12–15], resulting in lower rates of tooth decay [16]. Chemical warfare can also cause damage to the salivary glands, leading to decreased saliva production and increased susceptibility to tooth decay [17]. Wars and conflicts increase the risk of refugees, migrants, and Internally Displaced Persons’ (IDPs) [18] oral health status [19–24].

Ironically, war can heighten the vulnerability of children to ECC [25] through various pathways, including compromised maternal health, suboptimal feeding practices, inadequate oral hygiene, limited access to fluoridated toothpaste and water, insufficient availability of caries prevention measures, and reduced utilization of dental services [26]. Wars and conflicts also amplify the likelihood of both intimate and non-intimate sexual violence, creating harmful family environments that further elevate the risk of ECC [27–32]. The experience of violence increases women’s susceptibility to mental health issues [33–35], which, in turn, contributes to children’s exposure to cariogenic diets, poor oral hygiene practices, and an elevated risk of ECC [36–38].

There is little known about the potential connections between conflict, violence, insecurity, and the risk of ECC. The objective of this scoping review was to systematically examine the available evidence concerning the associations between ECC and elements such as peace, war, conflict, violence, justice, and strong institutions, as delineated in SDG 16. By mapping this evidence, we aimed to identify research gaps and provide recommendations to enhance the understanding of the relationship between SDG 16 and ECC.

## Methods

A scoping review was conducted to investigate the connections between ECC and the status of refugees, migrants, and IDPs [13, 39–82]. Additionally, we identified any potential gaps in the literature concerning the relationship between ECC and SDG 16 [83, 84]. The scoping review adhered to the Preferred Reporting Items for Systematic Reviews and Meta-Analyses Extension for Scoping Reviews (PRISMA-ScR) guidelines [85] to ensure methodological rigor and transparency.

### Research questions

This review aimed to address two key questions: (1) What is/are the existing evidence on the association between refugees, migrants, and internally displaced persons and ECC status? (2) What is/are the existing associations between ECC and maternal and child exposure to physical and sexual abuse, insecurity, crime, exploitation, torture, and displacement? These questions formed the foundation for exploring the relevant literature and gathering insights on these important topics.

### Search strategy

In January 2023, initial searches were conducted in PubMed, Web of Science, and Scopus to retrieve relevant literature. The search terms and strategy employed are detailed in Appendix 1. It should be noted that no specific protocol was published for this review.

### Eligibility criteria and article selection

For this review, only publications written in English up until January 2023 were included. Any type of published manuscript that presented findings related to the association between ECC and factors such as war, displacement, refugee and migrant status, violence (verbal, physical, or psychological) experienced by mothers, and adverse childhood experiences and child abuse was considered. The inclusion criteria required the study population to include children below the age of 72 months, and the publications had to have full texts available from which all relevant information could be extracted. Included publications encompassed letters, reviews, observational studies, and experimental studies. On the other hand, publications in the form of books and grey literature were excluded from this review.

### Data extraction

The data extraction process consisted of four phases. In the first phase, IA conducted a search in the three databases and imported the articles into the reference management software Mendeley®. The second phase involved removing duplicate papers, which was done by the same researcher. During the third phase, IA and AN independently reviewed the titles, abstracts, and full articles to assess their suitability for inclusion. Additionally, the reference lists of the screened publications were manually searched for any relevant articles. Any discrepancies in the selection of studies were resolved through consensus among IA, AN, and MOF. In the fourth phase, the results were shared with two subject experts (MET and AA) for their review. Publications were retained when there was consensus among the experts and the initial reviewers. The final consensus document was then shared with members of the Early Childhood Caries Advocacy Group (www.eccag.com) for further review. In case of any disagreements regarding the inclusion or exclusion of studies, the two experts and the three initial reviewers collaborated to reach a resolution. No additional sources were identified through contact with authors or institutions.

### Data charting

We created a data extraction form to capture relevant information from the selected publications. The extracted data encompassed details such as the publication year, study design, journal type (dental or non-dental), and the country where the study was conducted. In the case of reviews, we noted the countries associated with the first or last authors. For papers focusing on war, conflict, migrants, and refugees, we additionally identified the countries of origin and destination for the displaced individuals, as well as the specific SDG16 target addressed by each paper. A comprehensive summary of the extracted data, along with the corresponding manuscript references, can be found in Table 1.

**Table 1:** Summary of eligible publications

Author (Publication year)	Location where study was conducted	Origin of the refugee	Study design	Sample size	Age of study participants	Type of violence	Study findings
Greene and Chisick (1995) [39]	USA		Case-control study	42 abused children 822 non-abused children	3–11 years	Abused child	Abused children were 5.2 times more likely to have untreated, decayed primary teeth than other children.
Jessee (1995) [40]	USA		Narrative review			Abused child	Not applicable
Greene et al. (1994) [41]	USA		Case-control study	30 abused children 873 non-abused children	5–13 years	Abused child	Abused children are eight times more likely to have untreated, decayed permanent teeth than non-abused children.
Takayama et al. (1998) [42]	USA		Cross sectional	749 children	0–18 years	Children in foster care	4% children aged 0–6 years had dental caries.
Valencia-Rojas et al. (2008) [43]	Canada		Cross sectional	66 children	2–6 years	Abused and neglected child	Abused and neglected children had higher levels of tooth decay than the general population of 5-year-olds ECC prevalence did not differ between children with different types of maltreatment
Lang et al. (2019) [44]	Canada		Scoping review		Varied by study	Abused mothers	A positive relationship between exposure to intimate partner violence and ECC reported though mechanisms not well studied
Smitt et al. (2018) [45]	Netherlands		Case report		4-year-old	Neglected child	An association between child neglect and dental caries was established.
Keene et al. (2015) [46]	UK		Case-control study	79 children with child protection plans and 79 controls	2–11 years	Children in foster care	Caries in the primary dentition of children with a child protection plan was 1.76 (95% CI: 1.44–2.15) higher than the control
Bhatia et al. (2014) [47]	UK		Systematic review		Varies by study	Neglected child	Failure/delay in seeking care was associated with adverse dental consequences were highlighted, differentiating dental caries from dental neglect is difficult
Lourenço et al. (2013) [48]	Brazil		Cross sectional	149 children	5 years	Neglected children	A trend towards association between caries experience and risk factors suggestive of neglect but association not statistically significant.
Kvist et al. (2018) [49]	Sweden		Case-control study	86 abused and neglected children and 172 matched controls	2–18 years	Abused and neglected children	There is a high prevalence of dental caries among Swedish children suspected of child abuse and neglect
Duda et al. (2017) [50]	Brazil		Case-control study	120 abused children 240 non-abused children	3–15 years	Abused children	Children who were victims of abuse had a significantly higher prevalence of missing primary teeth (P = 0.04)
Harris (2018) [51]	UK		Narrative review			Abused children	Not applicable
Gurunathan and Shanmugaavel (2016) [52]	India		Cross sectional	478 pairs of parents and children	3–12 years	Neglected children	A significant higher DMFT (P = 0.003), deft (P = 0 < 0.001), pufa (P = 0.011) scores were seen in the higher dental neglect group.
Sillevis Smitt et al. (2017) [53]	Netherlands		Cross sectional	376 children	2–17 years	Abused and neglected children	A strong association between severe dental caries and child abuse and neglect. Severe dental caries could be regarded as an early symptom of child abuse and neglect.
Folayan et al. (2020) [54]	Multi-country		Ecological		0–5 years	Abused mothers	None of the indicators for violence against women was significantly associated with the prevalence of ECC.
Tokue et al. (2022) [55]	Japan		Case report		5-year-old	Abused children	Whole-body computed tomography for child abuse screening showed unnatural fracture in left arm and several dental caries
Simon et al. (2021) [56]	USA		Cross sectional	41,294 children and adolescents	0–17 years	Adverse childhood experience	Financial hardship (AOR: 1.85), caregiver divorce (AOR: 1.87), neighborhood violence (AOR: 2.09), and drug and alcohol problems (AOR: 2.11) were associated with caries
Kiatipi et al. (2021) [57]	Multi-country		Narrative review			Neglected children	Not applicable Dental neglect can be a part of a child’s general neglect with short-term complications, such as caries
Shmerling et al. (2020) [58]	Australia		Cross sectional	200 children	0–18 years	Children in foster care	40% of in children in foster care, residential care and kinship care had dental caries
Kopycka-Kedzierawski et al. (2022) [59]	USA		Cohort	189 children	1–3 years	Adverse childhood Experience	After controlling for all variables, no significant association between ECC free survival and parental alcohol use, depression, household disorganization, conflicts, stressful life events, anxiety and worry
Folayan et al. (2022) [60]	Multi-country		Ecological study		3–5 years	Abused mothers	For every 1% higher prevalence of emotional violence, there was 0.28% higher prevalence of ECC, and for every 1% higher percentage of physical violence, there was 0.21% higher prevalence of ECC. On the contrary, for every 1% higher prevalence of sexual violence, there was 0.35% lower prevalence of ECC prevalence.
Scheutz et al. (1983) [61]	Malaysia	Vietnam	Cross sectional	361 refugees	0 and 5 years	Refugees	dmft was 1.3 for 0-2-yr-olds, 7.4 for 3-5-yr-olds, 2,4 for 6-9-yr-olds and between 8.5 and 10.10 for the older age groups.
Todd and Gelbier (1990) [62]	UK	Vietnam	Cross sectional	268 Vietnamese children	0–19 years	Refugees	72.9% of the children 0–4 years old had caries with a mean dmft (standard deviation) of 4.46 (0.71) and a dmfs (standard deviation) of 8.96 (2.02). The caries experience in the primary teeth was higher for those who had spent longer time in Britain.
Cote et al. (2004) [63]	USA	Multiple countries	Case control	224 (121 Africa, 59 Eastern Europe, 44 others) newly arrived refugees 11,296 USA children (historical control)	6 months − 18 years	Refugees	51.3% of refugees had caries (38.0% of refugees from Africa, 79.7% of those from Eastern Europe and 50.0% of those from other nations had caries) while 49.3% of children from USA had caries (p = 0.55) 16.5% of refugees from Africa, 30.5% of those from Eastern Europe and 15.9% of those from other nations had ECC. Black refugees had significantly higher odds of having untreated caries than white children from USA (OR: 2.03; 95% CI: 1.40–2.95). White/other refugees had significantly higher odds of having untreated caries than white children from USA (OR: 9.43; 95% CI: 6.06–14.7).
Noaman et al. (2019) [64]	Iraq	Iraq	Cross sectional	79 pre-schoolers and their 79 mothers	4–5 years	Internally displaced persons	63% of the children had dental caries: 51.2% of 4-year-olds and 77.8% of 5-year-olds. Also, the dmft of 4-year-olds was 2.37 ± 3.33 while the dmft of 5-year-olds was 3.55 ± 3.79
Moreau et al. (2019) [65]	Canada	Multiple countries	Case control	2120 refugees and 117 Canadian children	1–14 years	Refugees	Refugee children had significant higher dmft/DMFT scores than Canadian children (7.29 ± 5.1 vs. 4.47 ± 5; p < 0.0001). Refugee status (OR = 5.08; 95% CI = 2.31–11.1) was significantly associated with caries experience.
Ogawa et al. (2019) [66]	USA	Multiple countries	Cross sectional	228 participants	2–5 years	Refugees	Most refugees were from Africa (44.3% or Asia (50.0%). More Asian refugees had a moderate or high caries risk (64% versus 44%) and need for urgent treatment (45.6% versus 30.7%) compared to Africans.
Hoover et al. (2017) [67]	Canada	Multiple countries	Cross sectional	33 recent immigrant and refugee children, and 86 adult guardians	3–15 year	Refugees and immigrants compared	Children of refugee had statistically significant higher decayed, missing, filled teeth scores (mean dmft/DMFT score 5.80 ± 4.24) than immigrant children (mean dmft/DMFT score 3.52 ± 3.78 (p < 0.001).
El Azrak et al. (2017) [68]	Canada	Multiple countries	Cross sectional	211 children	0–71 months	Refugees and immigrants combined	Overall, 45.5% of the children had ECC and 31.8% had severe ECC. Increasing age, the presence of debris on teeth, parents believing their child has dental problems and the presence of enamel hypoplasia were significantly and independently associated with ECC and severe ECC
Reza et al. (2016) [69]	Canada	Multiple countries	Scoping review		0–18 years	Refugees and immigrants (new comers) combined	When compared with children of Canadian-born parents, children of newcomers presented higher mean def in the primary teeth (3.05 vs. 1.83, p < 0.05) and mean DMF in the permanent teeth (0.73 vs. 0.42, p < 0.05). In the United States, compared with children of USA-born parents, children of immigrants had a significantly larger number of carious surfaces (11.5 vs. 9.4, p = 0.01) and twice the prevalence of ECC (OR: 2.06; 95% CI: 1.47–2.88). Children of refugee children had greater number of untreated caries (up to about 75%)
Nicol et al. (2015) [70]	Australia	Multiple countries	Cross sectional	105 children	3–5 years	Refugees (humanitarian entrants and asylum seeking)	62% had caries. After adjustment for age, gender and total number of teeth, caries incidence was significantly associated with BMI-for-age Z score (p = 0.02).
Francis et al. (2012) [71]	Australia	Unspecified	Letter to editor			Refugees	Not applicable
Quach et al. (2015) [72]	Australia	Multiple countries	Cross sectional	350 patients	0–18 years	Refugees and immigrants combined	46.1% had visible caries and 51.6% had caries experience (dmft/DMFT > 0). African-born children were less likely to have caries compared to other overseas-born children (adjusted PR 0.73, 95% CI: 0.58–0.93).
Lauritano et al. (2021) [73]	Multi-country	Multiple countries	Systematic review		Varies by study	Refugees, migrants and asylum seekers	Higher prevalence of caries experience among migrant groups compared with the non-migrant population.
Flynn et al. (2021) [74]	USA	Somali	Cross sectional	267 children 99 mothers	6 months to 12 years	Mother and child refugees	dmft for children > 2 years was 0%, for 2–5 years was 2.3 (6.1); for 6-11-years was 4.2 (8.2) and for 12 years was 0.8 (1.2). Each additional mother’s DMFS was associated with a 1.6% increase in the mean number of dfs for her child (95% CI 0.1–3.2%)
Al-Ani et al. (2021) [75]	Germany	Multiple countries	Cross sectional	544 refugees	3–75 + years	Refugees	3-year-old refugees had dmft of 2.62 ± 3.6 compared with 0.48 dmft in the German resident population. The dt was 2.54 ± 3.6, mt was 0.05 ± 0.3 and the ft was 0.03 ± 0.2. 16% had a pufa index greater zero
Alrashdi et al. (2021) [76]	USA	Multiple countries	Randomised control trial	100 interventions and 100 controls	0–12 years	Parents and children refugees	The DMFT/dmft score increased from baseline to six months after educational intervention (0.28; 95% CI: 0.06, 0.50; *p* = 0.012) and did not differ significantly from those who sis not receive intervention (β = −0.23, 95% CI: (− 0.57, 0.11; *p* = 0.18)
Bhusari et al. (2020) [77]	Multi-country	Multiple countries	Systematic review		Varies by study	Refugees	Caries prevalence ranged between 50% and 100%. Prevalence was proportional to age, inversely associated with education and not significantly associated with gender and country of origin
Werneck et al. (2008) [78]	Canada	Multiple countries	Case-control	52 ECC cases and 52 controls		Refugees	mean (SD) age = 32.4 (11) months The strongest predictors of ECC in this immigrant population were lack of dental care (AOR = 3.96, 95%CI: 1.34, 11.70) and lack of dental insurance (AOR = 4.87, 95%CI: 1.85, 12.82).
Joury et al. (2021) [81]	Lebanon	Syria	Cross sectional	823 Syrian refugee schoolchildren	4–15 years	Refugees	Prevalence of caries was 90%. Children in protracted displacement were significantly more likely to have a higher number of decayed teeth compared to their counterparts who had been displaced for less than five years (RR = 1.19; 95% CI = 1.09–1.29; P < 0.001).
Zinah and Al-Ibrahim (2021) [80]	Multi-country	Multiple countries	Scoping review		Varies by study	Refugees	The levels of diseases were always higher for refugees compared to levels reported for the wider populations of the host countries
Toverud (1949) [13]	Norway	No origin and destination countries	Repeated cross sectional	600–700 children in years 1939, 1944–1948	2.5-7 years	War	Decrease in caries frequency during the war. Decrease may be attributed to the lowering in consumption of refined carbohydrates and the increase in consumption of more natural protective foods.
Joury (2019) [81]	Syria	No origin and destination countries	Narrative review		3–5 years	War	ECC prevalence increased from 50% in 1991 to 56% in 2011, with a dmft value of 6.1 for 3-year-olds; and from 74% in 1991 to 81% in 2011 with a dmft value of 8.6 for 5-year-olds.
Folayan et al. (2020) [82]	Multi-country	No origin and destination countries	Ecological		3–5 years	Political instability and terrorism	Political stability/absence of terrorism (β = 0.40) was directly associated with a higher ECC prevalence. Control of corruption (β = − 0.23) was indirectly associated with a lower ECC. Political stability/absence of terrorism (β = 0.34) was one of two factors with the greatest effects on ECC prevalence

**Fig. 1:**
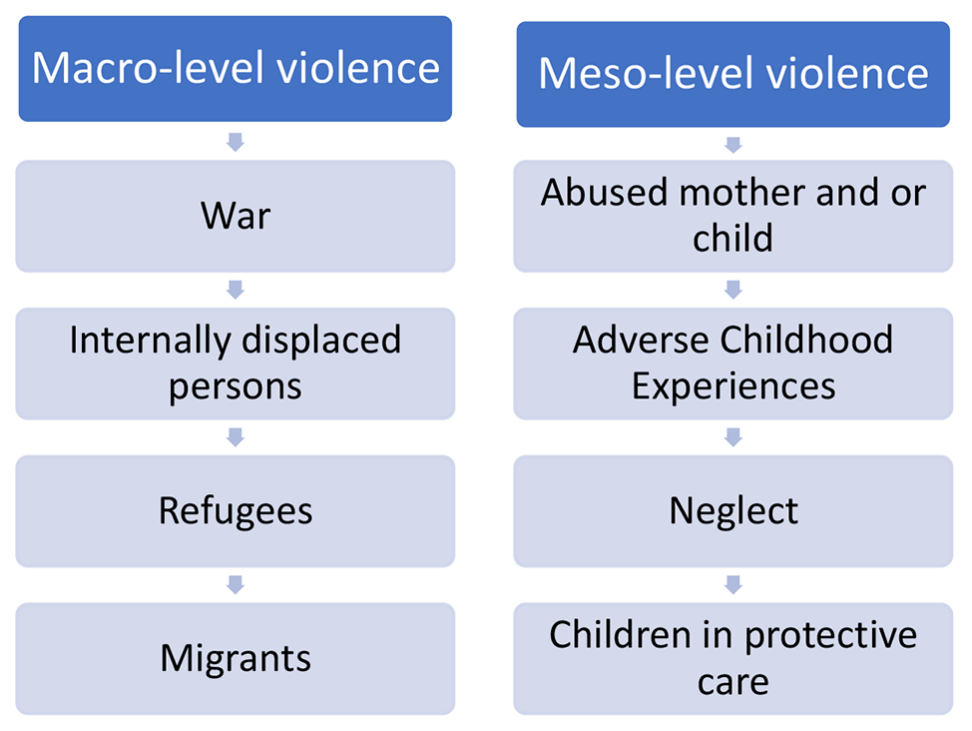
Categorization of papers included in the study

We classified the target population into two categories: those who were affected by the consequences of peace disruption at the macro-level (refugees, migrants, and IDPs) and those who experienced the effects of violence at the meso-level (child neglect, abuse of mothers or children, children in protection services, and those who had adverse childhood experiences). We examined the documented associations between ECC and these macro- and meso-level risk factors. The categorization of the papers included in the study is illustrated in Fig. 1.

### Analysis

Descriptive statistics, including frequencies and percentages, were utilized to present the data in this study. To visually represent the flow of data from one set of entities to another, we employed a Sankey diagram [86]. The Sankey diagram is particularly useful in illustrating the movement of displaced individuals from their country of origin to their destination countries, as observed in the papers examining the ECC situation among refugees and migrants. The countries were categorized according to the WHO regions [87], which include the Americas Regions (AMR), Eastern Mediterranean Region (EMR), African Region (AFR), European Region (EUR), Southeast Asian Region (SEAR), and Western Pacific Region (WPR).

## Results

**Fig. 2:**
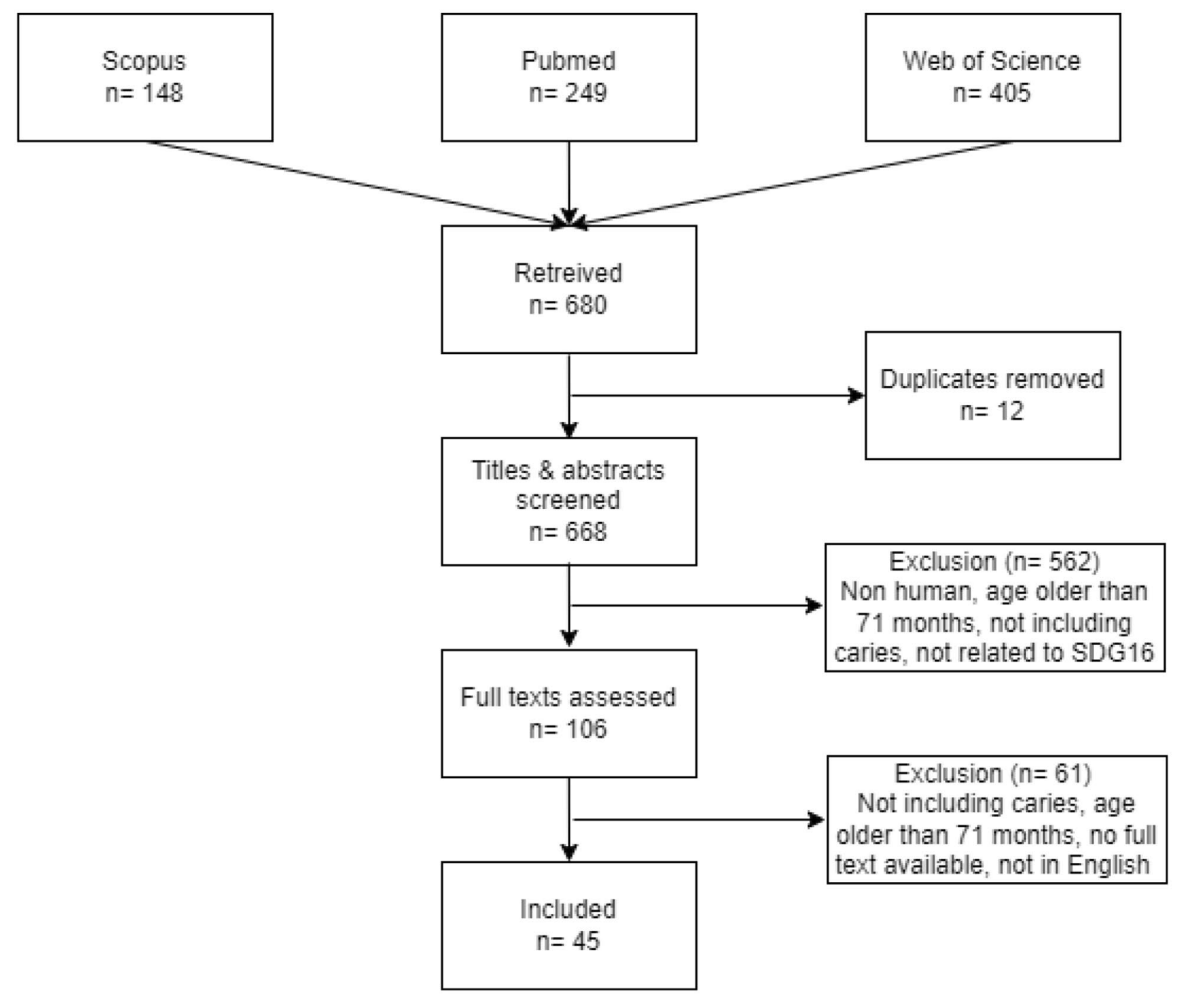
Flowchart of study selection process

As shown in Fig. 2, the initial search of PubMed, Web of Science, and Scopus databases resulted in 680 potentially relevant publications. After removing duplicates, 668 publications remained for screening based on their titles, abstracts, and full texts. Out of these, 623 publications were excluded based on the predefined criteria, resulting in a final selection of 45 publications for inclusion in the analysis.

**Fig. 3:**
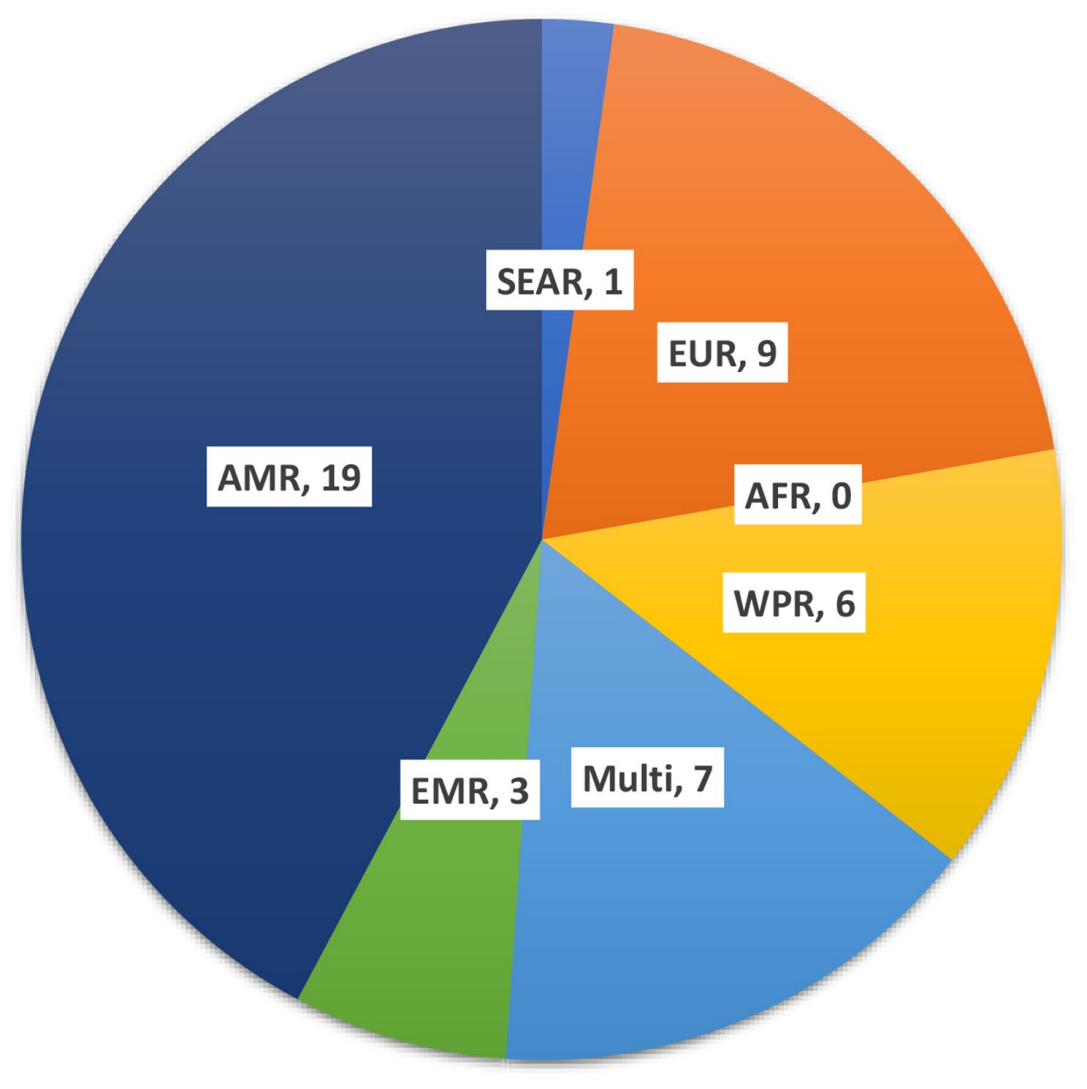
Distribution of included studies by World Health Organisation region of country of study or authors (AMR: Americas Regions, EMR: Eastern Mediterranean Region, AFR: African Region, EUR: European Region, SEAR: Southeast Asian Region, WPR: Western Pacific Region)

Figure 3 displays the regions where the studies were conducted. Many of the studies (42.2%) were from the AMR, including ten studies from the USA [39–42, 56, 59, 63, 66, 74, 76], seven studies from Canada [43, 44, 65, 67–69], and two studies from Brazil [48, 50]. Following that, there were nine studies (20%) from EUR, consisting of four studies from the United Kingdom [46, 47, 51, 62], two studies from the Netherlands [45, 53], one study from Sweden [49], one study from Germany [75], and one study from Norway [13]. Additionally, there were six studies (8.3%) from the WPR, including four studies from Australia [58, 69–72], one study from Japan [48], and one study from Malaysia [61]. The EMR had three studies (6.7%) - one from Iraq [64], one from Lebanon [79], and one from Syria [81]. There was one study (2.2%) from the SEAR, specifically India [52]. Finally, there were seven multi-country studies (15.6%) [54, 60, 63, 73, 77, 80, 82]. Collectively, the studies from the AMR and EUR accounted for 62.2% of all the papers, while no studies were identified from the AFR region.

**Fig. 4:**
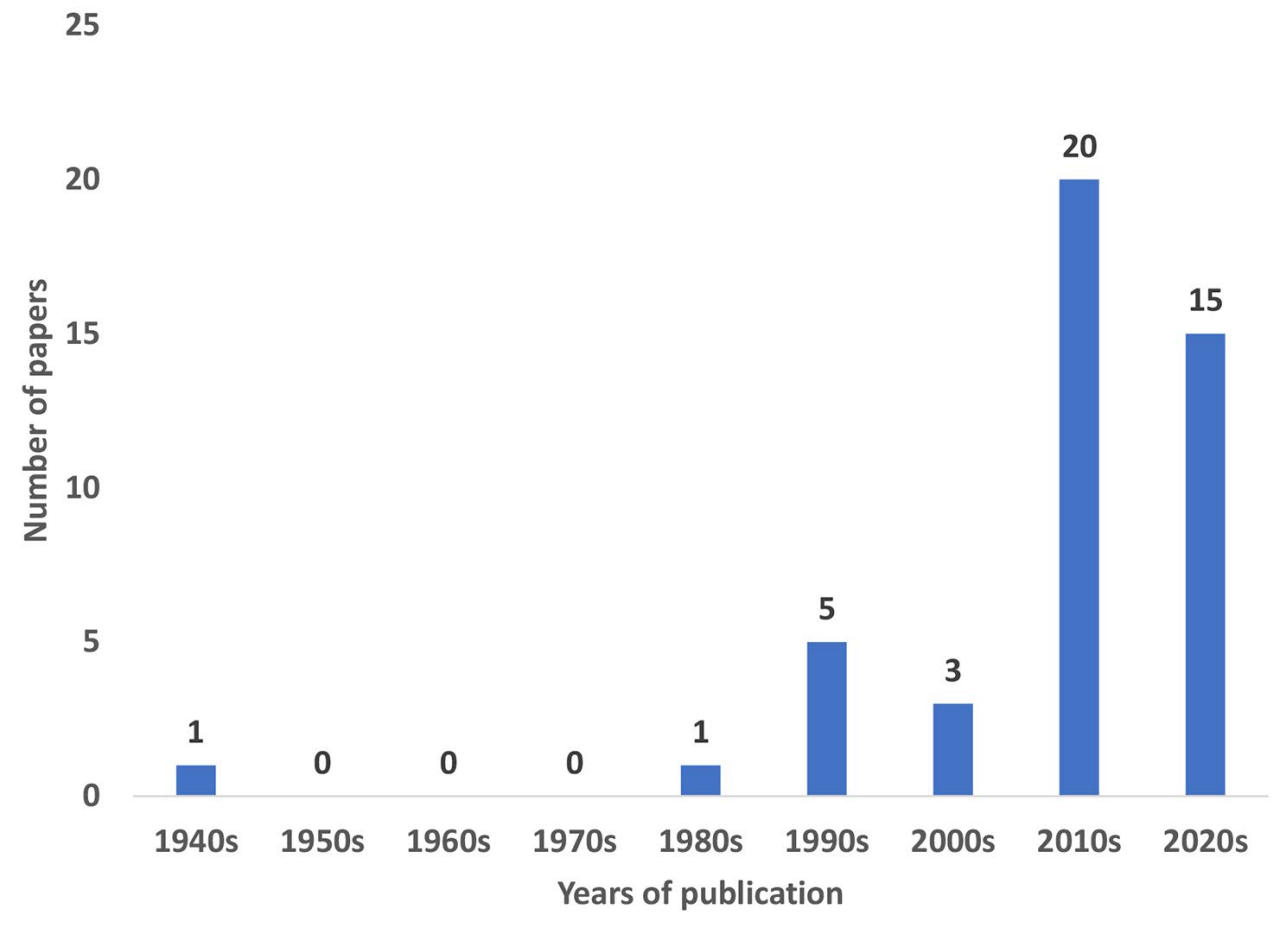
Number of papers on ECC and SDG 16 related topics over time

Figure 4 illustrates that nearly 30 years passed between the publication of the first and second articles exploring the association between ECC and SDG 16. Furthermore, 35 articles (77.8%) were published after 2010, coinciding with the decade when the SDGs were introduced. The majority of articles (46.7%) focused on abuse, violence, and neglect as risk factors for ECC [39–41, 43–45, 47–55, 59, 61, 67]. The most frequently studied populations (44.4%) were migrants, refugees, and displaced individuals [61–80].

Three articles (6.7%) explored the relationship between war, political instability, and terrorism and the prevalence of ECC [13, 81, 82]. The first article focused on investigating the impact of World War II on ECC prevalence among Norwegian children in 1940 [13]. The second article examined the effect of the war in Syria on ECC prevalence among Syrian children [81]. The third paper explored the influence of political instability and terrorism on ECC prevalence across multiple countries [82].

Twenty-three articles (51.1%) were published in dental journals [41, 43, 44, 46, 47, 50, 52–55, 57, 59, 60, 62, 66, 68–70, 72, 75, 79, 82], while 22 articles (48.9%) were published in non-dental journals. These articles covered a wide range of topics, including public health, family health, child health, child abuse and neglect, and migrant health [13, 39, 40, 42, 45, 48, 49, 51, 56, 58, 63–65, 67, 73, 74, 76–78, 80, 81].

There were 18 (40.0%) cross-sectional studies [42, 43, 48, 52, 53, 56, 58, 62, 64, 66–68, 70, 72, 74, 75, 79, 80] and seven (15.6%) case-control studies [39, 41, 46, 49, 50, 63, 65]. Additionally, there was one study with a repeated cross-sectional design [13], three ecological studies [54, 60, 82], one case-control study [78], one cohort study [59], two case reports [45, 55], four narrative reviews [40, 51, 57, 81], and one letter to the editor [71]. Furthermore, there were three scoping reviews [44, 69, 80], three systematic reviews [47, 73, 77], and one randomized clinical trial [76].

Among the 22 articles exploring the links between meso-level factors and caries, seven examined the associations between caries and child abuse [39–41, 43, 50, 51, 55], five focused on caries and child neglect [45, 46, 48, 51, 57], two investigated caries in children exposed to abuse and neglect [53, 56], three explored caries in children in foster care [42, 46, 58], two examined the relationship between adverse childhood experiences and caries [56, 59], and three investigated the impact of maternal exposure to violence as risk factors associated with ECC [44, 54, 60].

**Fig. 5:**
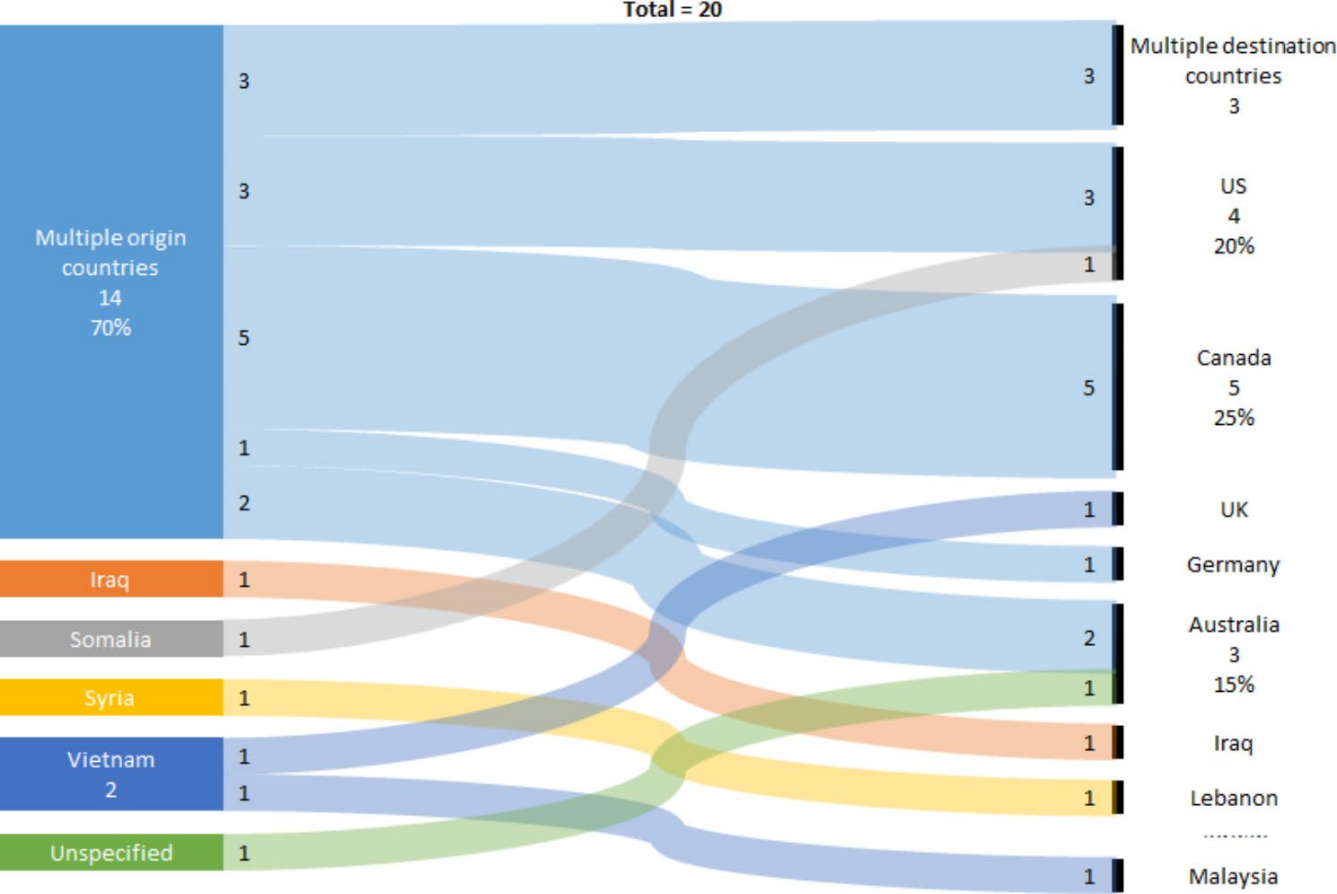
Sankey diagram of flow of displaced people in studies on ECC in migrants and refugees by origin and destination countries

Figure 5 illustrates the migration patterns of displaced individuals from their origin countries to destination countries as depicted in the 20 papers examining ECC in refugees and migrants. Among these papers, fourteen (70%) focused on describing the caries profile of children from various countries of origin who resettled in Canada [65–69, 78], the USA [63, 66, 76], Australia [70, 72], Germany [7], or multiple other countries [73, 77, 80]. Two papers examined the caries profile of children from Vietnam seeking refuge in either the UK [61] or Malaysia [62]. Additionally, two papers investigated the caries profile of children from EMR countries, specifically Syrian refugees in Lebanon [79] and Somali refugees in the USA [74]. One study explored the caries profile of internally displaced persons (IDPs) from Iraq [64], another examined migrants [78], and six studies examined both refugees and migrants [67–70, 72, 73].

All the studies examining the status of IDPs, refugees, and migrants [13, 59–80] indicate that forced displacement is likely to be associated with a higher prevalence [62, 63, 65, 67, 69, 73, 77] and severity of caries [59–62, 64–66, 68, 75], particularly untreated caries [62, 68]. Moreover, forced displacement is also linked to a high prevalence and severity of caries in the primary dentition [75]. The duration of stay appears to affect the risk of caries among refugees, as children of new immigrants exhibit a higher prevalence and severity of ECC [13, 68, 79]. Additionally, the risk of caries may vary depending on the country of origin [62, 72]. For instance, refugees from Africa have a lower prevalence of caries compared to other refugee groups, while refugees from Vietnam who have resided in Britain for an extended period exhibit a higher prevalence of caries compared to newly arrived refugees. Caries prevalence and severity may also differ based on race [63, 66], as black and white/other refugees tend to have more untreated caries than white children in the US, and black refugees demonstrate a lower prevalence of caries compared to Asians.

Thirteen studies (28.9%) specifically focused on caries in the primary teeth of children under the age of 6 years (early childhood caries, ECC) [45, 48, 54, 59–62, 64, 66, 68, 70, 81, 82]. Most of these studies (except for three) indicated an association between meso-level factors and a higher risk of ECC. However, Folayan et al. [54, 60] found no statistically significant association between intimate partner violence and other forms of violence experienced by women and ECC, while Kopycka-Kedzierawski et al. [59] found no association between ACE and ECC. Among IDPs, refugees, and migrants, the prevalence of ECC may not always be higher than that of the host community [63, 71], but the prevalence of untreated caries may be higher [66, 69]. Regarding the impact of war, the findings appeared paradoxical. While a study from the 1940s among Norwegian children suggested that war was associated with a lower prevalence of ECC [80], a study among children in Syria indicated that war was associated with an increase in the prevalence of ECC [81], and an ecological study [82] suggested that wars and conflicts were associated with a higher risk of ECC.

**Fig. 6:**
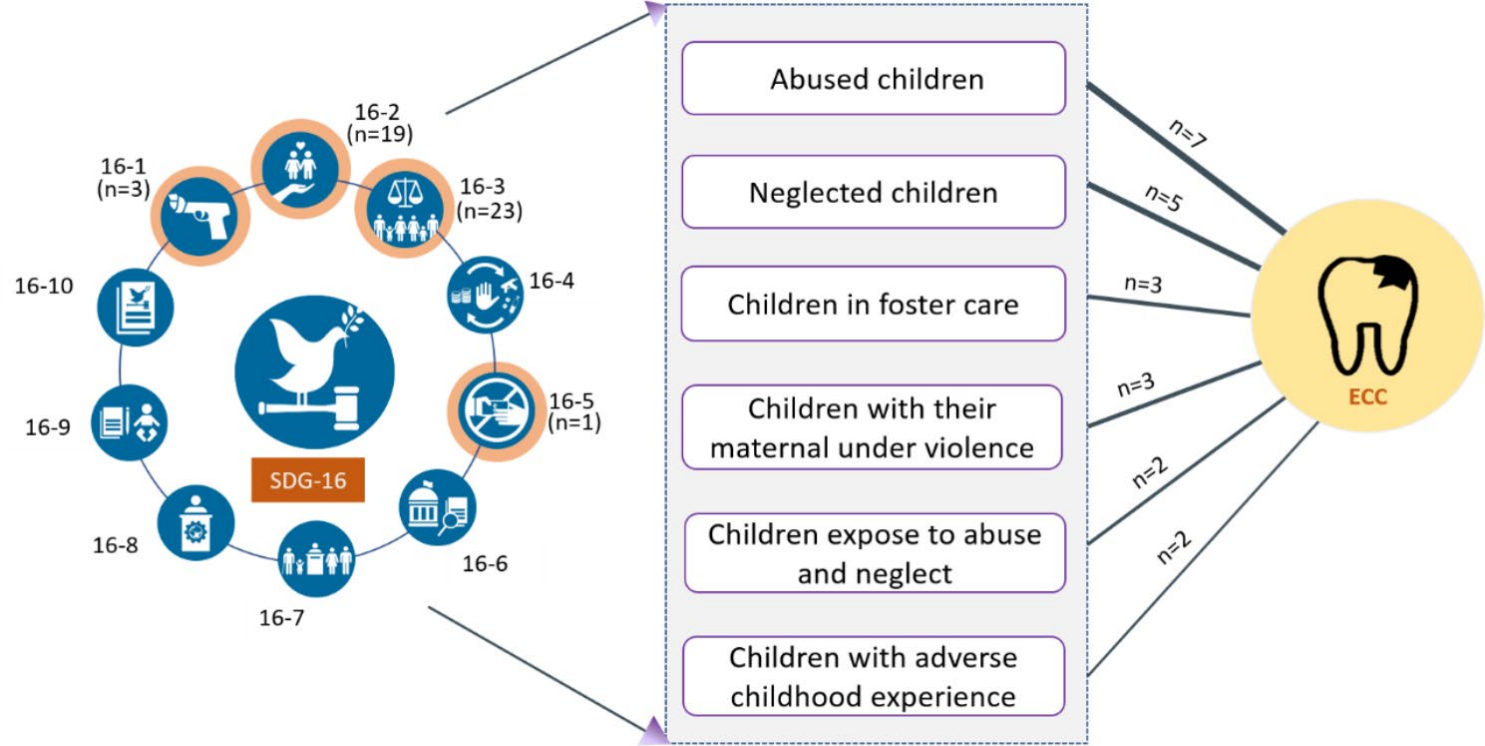
The conceptual framework of early childhood caries and peace (SDG 16) 16 − 1 Reduce violence everywhere 16 − 2 Protect children from abuse, exploitation, trafficking and violence 16 − 3 Promote the rule of law and ensure equal access to justice 16 − 4 Combat organized crime and illicit financial and arms flows 16 − 5 Substantially reduce corruption and bribery 16 − 6 Develop effective, accountable and transparent institutions 16 − 7 Ensure responsive, inclusive and representative decision-making 16 − 8 Strengthen the participation in global governance 16 − 9 Provide universal legal identity 16 − 10 Ensure public access to information and protocol fundamental freedoms

Figure 6 provides a visual representation of the relationship between SDG 16 and ECC. Most of the studies included in the analysis focused on specific targets within SDG 16. Specifically, most studies addressed SDG16.1, which aims to reduce all forms of violence [44, 54, 60]. Additionally, there were several studies that examined SDG16.2, which aims to end all forms of abuse, violence, and torture of children [39–43, 45–53, 55–59]. Furthermore, some studies explored SDG16.3, which aims to promote the rule of law and ensure justice for all [13, 61–82]. Only one study specifically addressed SDG16.5, which aims to substantially reduce corruption and bribery [82].

## Discussion

The results of this scoping review reveal a diverse range of studies examining the association between SDG 16 and oral health, but only a limited number of studies specifically focusing on the relationship between SDG 16 and ECC. Most of the studies emphasized the meso-level determinants of ECC, such as ACEs, abuse, and neglect, while fewer studies investigated the macro-level determinants of ECC. It is noteworthy that most of the studies were conducted in resource-rich settings and primarily involved populations of refugees, migrants, and IDPs. Furthermore, most of the studies were observational in nature, with only one clinical trial conducted in the United States examining the impact of education on reducing the risk of caries among children with refugee status. Notably, there were no studies identified that specifically assessed the association between ECC and SDG 16.4, 16.6–16.10, 16 A, and 16B.

This scoping review represents the first comprehensive analysis examining the specific connection between SDG 16 and ECC. Our initial evaluation of the available research literature on the relationship between SDG 16 and ECC indicates that factors such as child abuse, neglect, being in protective care, and having refugee/migrant/IDP status are associated with a higher prevalence and severity of ECC. However, the association between maternal abuse and ECC showed no significant correlation, although a trend was observed. Additionally, the relationship between adverse childhood experiences (ACE), war, and ECC remains unclear, and further research is needed to determine the magnitude of the impact of SDG 16 indicators on the prevalence and severity of ECC.

Furthermore, the studies suggest that individuals with refugee, migrant, and IDP status are at increased risk for ECC. Refugees are individuals who leave their home countries due to threats to their safety, lives, and human rights violations, while migrants seek opportunities for work, education, or to escape poverty, natural disasters, or political instability, without facing the same risks as refugees [10]. Refugees and migrants cross international borders, whereas IDPs relocate within their own country without crossing an international boundary [10]. A study reported that differences in the status of those who were forced to move, can affect their access to care and expose them to distinct physical and psychological threats [88]. However, the existing research evidence lack sufficient information about the link between different populations of refugees, migrants, and IDPs.

In 2022, the global number of forcibly displaced persons reached approximately 100 million, with IDPs accounting for up to 53.2 million individuals [89]. Surprisingly, we only identified one study investigating ECC among IDPs in Iraq [64]. Additionally, there were no studies on ECC among refugees from significant origin countries such as Venezuela, Ukraine, Afghanistan, and South Sudan, which collectively account for 50% of all current global refugees [90]. Moreover, no studies on ECC among refugees in Turkey, Colombia, Pakistan, and Uganda were found, despite these countries hosting 28% of the refugee population [90]. Furthermore, no studies from Africa were available. Low- and middle-income countries, where a significant population of IDPs, migrants, and refugees reside, are already grappling with a growing burden of oral diseases [91] and struggling with inadequate oral health systems to address this burden [92]. Therefore, there is a critical need for studies that explore the risk, magnitude, and variations in ECC among large populations of IDPs compared to similar populations in their country of origin or host countries. Such data would be invaluable in improving the planning and implementation of population-specific oral health interventions for IDPs.

Moreover, most of the available evidence supports the association between abuse, neglect, violence, and conflict with an increased prevalence and severity of ECC. A detailed analysis revealed that children who experience physical or sexual abuse are at a higher risk of untreated caries [43]. However, the association between maternal exposure to intimate partner violence and ECC risk remains unclear, although a positive but non-significant trend was observed [44, 54, 60]. A prospective cohort study conducted in New York, USA, over a period of two years found no significant association between family conflict and disorganization with the onset of ECC [59]. Conducting a meta-analysis of the existing evidence would be valuable in assessing the strength of the current findings and identifying any gaps that need to be addressed.

Our study highlights that most studies examining meso-level factors associated with ECC are predominantly conducted in resource-rich settings, despite the higher risk of abuse, neglect, and ACE in low and middle-income countries [93]. It is worth noting that low and middle-income countries are less likely to report on three out of the seven strategies proposed by the World Health Organization (**I**mplementation of laws; **N**orms change; **S**afe environments; **P**arental support; **I**ncome strengthening; **R**esponse services provision and **E**ducation - INSPIRE) to address child abuse [94]. The failure to implement comprehensive programs and practices to address child abuse may have a detrimental impact on ECC control, as exposure to violence can be a contributing factor to ECC.

Further research is warranted to investigate the potential impact of other SDG indicators on ECC. For instance, SDG 16.4 aims to reduce financial and arms flow while strengthening the return of stolen assets. Evidence suggests that investments in arms and the misappropriation of country assets divert funds away from education and health sectors, resulting in direct and indirect negative effects on children’s health [95–98]. However, currently, there is no available evidence on how these resource diversions specifically impact the risk of ECC. Conducting studies to assess the influence of such policies on disease profiles presents challenges, yet they are crucial to strengthening the rule of law and reducing investments in arms [99–101].

Novel methodologies should be explored to establish the link between other SDG 16 indicators and ECC. These studies would provide valuable evidence to support policies that promote peace and ensure access to oral care services for infants, toddlers, and pre-school children, thereby reducing the risk of ECC. Additionally, it is important to acknowledge the resilience and resourcefulness of migrants as they adapt to new environments. Exploring these strengths to inform strategies aimed at enhancing access to protective factors and buffering the impact of the risk factors for ECC among children in migrant populations.

In conclusion, ECC is a complex condition influenced by various social determinants, including factors associated with humanitarian crises, as emphasized in this study. It is crucial for humanitarian organizations, social activists, and health institutions to implement community-based initiatives and oral health promotion programs that specifically target children who are migrants, refugees, or residing in displaced centres. Furthermore, further research is necessary to investigate how forced movements contribute to the risk of ECC in infants, toddlers, and pre-school children, and other pathways linking the United Nations’ SDG 16 and ECC. This knowledge will enable the development of effective strategies to mitigate the detrimental impact of ECC on the growth and development of these children.

### Electronic supplementary material

Below is the link to the electronic supplementary material.

Supplementary Material 1

## Data Availability

All data generated or analysed during this study are included in this published article and its supplementary information.

## Abbreviations

AOR: Adjusted Odds Ratio
CI: Confidence Interval
ECC: Early Childhood Caries
dmft: Decayed, Missing and Filled primary Teeth
DMFT: Decayed, Missing and Filled permanent Teeth
OR: Odds Ratio
PRISMA-ScR: Preferred Reporting Items for Systematic Reviews and Meta-Analyses Extension for Scoping Reviews guidelines
pufa: Pulpal involvement Ulceration Fistula Abscess
PR: Prevalence Ratio
SD: Standard Deviation
SDG: Sustainable Development Goal
USA: United States of America
