# Supplementary material for: A scoping review linking early childhood caries to violence, neglect, internally displaced, migrant and refugee status
Source: BMC Oral Health. 2023 Oct 11;23:747. doi: 10.1186/s12903-023-03459-0 (PMC10568772; doi:10.1186/s12903-023-03459-0)
Supplement: Supplementary file 1 — Supplementary Material 1 [file 12903_2023_3459_MOESM1_ESM.docx]

Appendix 1; Search strategies for Pubmed, Scopus, WOS and the Figures

**Pubmed search strategy**

| Search # | Query | Search Details | Results |
| --- | --- | --- | --- |
| 3 | "Armed Conflicts"[Mesh] OR "War Crimes"[Mesh] OR "Warfare"[Mesh] OR "Democracy"[Mesh] OR "Government"[Mesh] OR "Crime"[Mesh] OR "Criminals"[Mesh] OR "Politics"[Mesh] OR "Genocide"[Mesh] OR "Homicide"[Mesh] OR "Human Trafficking"[Mesh] OR "Refugees"[Mesh] OR "Violence"[Mesh] OR "Domestic Violence"[Mesh] OR "Gender-Based Violence"[Mesh] OR "Intimate Partner Violence"[Mesh] OR "Physical Abuse"[Mesh] OR "Torture"[Mesh] OR "Human Rights"[Mesh] OR "Human Rights Abuses"[Mesh] OR "Child Abuse"[Mesh] OR "Freedom"[Mesh] OR "Geneva Convention" OR peace OR corruption OR bribery OR "justice system" OR "good governance" OR "political instability" AND "Dental Caries" [Mesh] | ("Armed Conflicts"[MeSH Terms] OR "War Crimes"[MeSH Terms] OR "Warfare"[MeSH Terms] OR "Democracy"[MeSH Terms] OR "Government"[MeSH Terms] OR "Crime"[MeSH Terms] OR "Criminals"[MeSH Terms] OR "Politics"[MeSH Terms] OR "Genocide"[MeSH Terms] OR "Homicide"[MeSH Terms] OR "Human Trafficking"[MeSH Terms] OR "Refugees"[MeSH Terms] OR "Violence"[MeSH Terms] OR "Domestic Violence"[MeSH Terms] OR "Gender-Based Violence"[MeSH Terms] OR "Intimate Partner Violence"[MeSH Terms] OR "Physical Abuse"[MeSH Terms] OR "Torture"[MeSH Terms] OR "Human Rights"[MeSH Terms] OR "Human Rights Abuses"[MeSH Terms] OR "Child Abuse"[MeSH Terms] OR "Freedom"[MeSH Terms] OR "Geneva Convention"[All Fields] OR ("peace"[All Fields] OR "peaceful"[All Fields] OR "peacefulness"[All Fields]) OR ("corrupt"[All Fields] OR "corrupted"[All Fields] OR "corrupting"[All Fields] OR "corruption"[All Fields] OR "corruptions"[All Fields] OR "corrupts"[All Fields]) OR "bribery"[All Fields] OR "justice system"[All Fields] OR "good governance"[All Fields] OR "political instability"[All Fields]) AND "Dental Caries"[MeSH Terms] | 468 |
| 2 | "Dental Caries"[Mesh] | "Dental Caries"[MeSH Terms] | 49,690 |
| 1 | "Armed Conflicts"[Mesh] OR "War Crimes"[Mesh] OR "Warfare"[Mesh] OR "Democracy"[Mesh] OR "Government"[Mesh] OR "Crime"[Mesh] OR "Criminals"[Mesh] OR "Politics"[Mesh] OR "Genocide"[Mesh] OR "Homicide"[Mesh] OR "Human Trafficking"[Mesh] OR "Refugees"[Mesh] OR "Violence"[Mesh] OR "Domestic Violence"[Mesh] OR "Gender-Based Violence"[Mesh] OR "Intimate Partner Violence"[Mesh] OR "Physical Abuse"[Mesh] OR "Torture"[Mesh] OR "Human Rights"[Mesh] OR "Human Rights Abuses"[Mesh] OR "Child Abuse"[Mesh] OR "Freedom"[Mesh] OR "Geneva Convention" OR peace OR corruption OR bribery OR "justice system" OR "good governance" OR "political instability" | "Armed Conflicts"[MeSH Terms] OR "War Crimes"[MeSH Terms] OR "Warfare"[MeSH Terms] OR "Democracy"[MeSH Terms] OR "Government"[MeSH Terms] OR "Crime"[MeSH Terms] OR "Criminals"[MeSH Terms] OR "Politics"[MeSH Terms] OR "Genocide"[MeSH Terms] OR "Homicide"[MeSH Terms] OR "Human Trafficking"[MeSH Terms] OR "Refugees"[MeSH Terms] OR "Violence"[MeSH Terms] OR "Domestic Violence"[MeSH Terms] OR "Gender-Based Violence"[MeSH Terms] OR "Intimate Partner Violence"[MeSH Terms] OR "Physical Abuse"[MeSH Terms] OR "Torture"[MeSH Terms] OR "Human Rights"[MeSH Terms] OR "Human Rights Abuses"[MeSH Terms] OR "Child Abuse"[MeSH Terms] OR "Freedom"[MeSH Terms] OR "Geneva Convention"[All Fields] OR "peace"[All Fields] OR "peaceful"[All Fields] OR "peacefulness"[All Fields] OR "corrupt"[All Fields] OR "corrupted"[All Fields] OR "corrupting"[All Fields] OR "corruption"[All Fields] OR "corruptions"[All Fields] OR "corrupts"[All Fields] OR "bribery"[All Fields] OR "justice system"[All Fields] OR "good governance"[All Fields] OR "political instability"[All Fields] | 560,112 |

Filters applied: English, Newborn: birth-1 month, Infant: birth-23 months, Infant: 1-23 months, Child: 6-12 years, Preschool Child: 2-5 years

Scopus search strategy

| TITLE-ABS-KEY ( ( {actual innocence} OR {false confession} OR {armed conflict} OR {armed conflicts} OR {civil conflict} OR {civil conflicts} OR ( war AND ( conflict OR warfare OR democracy OR {Geneva Convention} OR treaty OR peace ) ) OR {peacekeeping} OR ( corruption AND ( {institution} OR {public official} OR {government} OR {bribery} OR {conflict} ) ) OR crime OR crimes OR criminal OR {democratic deficit} OR ( democrati?ation AND ( institutional OR conflict OR decision-making OR society OR politics OR {financial aid} ) ) OR {ethnic conflict} OR {ethnic conflicts} OR exoneration OR genocid* OR homicid* OR murder* OR {human trafficking} OR {criminal justice system} OR {justice system} OR {arbitrary justice} OR refugee* OR terroris* OR violence OR torture OR {effective rule of law} OR {arms flow} OR {transparent institution} OR {transparent institutions} OR {good governance} OR {legal identity for all} OR {freedom of information} OR {human rights institution} OR {human rights activists} OR {fundamental freedom} OR {fundamental freedoms} OR {violent conflict} OR {violent conflicts} OR {peaceful society} OR {effective institution} OR {effective institutions} OR {accountable institution} OR {accountable institutions} OR {inclusive institution} OR {inclusive institutions} OR {child abuse} OR {arbitrary detention} OR {unsentenced detention} OR {judicial system} OR {criminal tribunal} OR {inclusive society} OR {inclusive societies} OR {responsive institution} OR {responsive institutions} OR {fair society} OR {fair societies} OR {legal remedy} OR {legal remedies} OR {independence of judiciary} OR {independent judiciary} OR {separation of powers} OR extremism OR {war crime} OR {peaceful society} OR {organized crime} OR {illicit transfer} OR {illicit money} OR {arms trafficking} OR {cybercrime} OR {insurgence} OR {democratic institution} OR {political instability} OR ( {political decision-making} AND ( responsive OR inclusive OR participatory OR representative ) ) OR {Aarhus Convention} OR {press freedom} OR {freedom of speech} ) AND NOT ( {disease} OR {genetics} ) ) | 709,287 |
| --- | --- |
| KEY (dental caries) | 63,950 |
| TITLE-ABS-KEY ( ( {actual innocence} OR {false confession} OR {armed conflict} OR {armed conflicts} OR {civil conflict} OR {civil conflicts} OR ( war AND ( conflict OR warfare OR democracy OR {Geneva Convention} OR treaty OR peace ) ) OR {peacekeeping} OR ( corruption AND ( {institution} OR {public official} OR {government} OR {bribery} OR {conflict} ) ) OR crime OR crimes OR criminal OR {democratic deficit} OR ( democrati?ation AND ( institutional OR conflict OR decision-making OR society OR politics OR {financial aid} ) ) OR {ethnic conflict} OR {ethnic conflicts} OR exoneration OR genocid* OR homicid* OR murder* OR {human trafficking} OR {criminal justice system} OR {justice system} OR {arbitrary justice} OR refugee* OR terroris* OR violence OR torture OR {effective rule of law} OR {arms flow} OR {transparent institution} OR {transparent institutions} OR {good governance} OR {legal identity for all} OR {freedom of information} OR {human rights institution} OR {human rights activists} OR {fundamental freedom} OR {fundamental freedoms} OR {violent conflict} OR {violent conflicts} OR {peaceful society} OR {effective institution} OR {effective institutions} OR {accountable institution} OR {accountable institutions} OR {inclusive institution} OR {inclusive institutions} OR {child abuse} OR {arbitrary detention} OR {unsentenced detention} OR {judicial system} OR {criminal tribunal} OR {inclusive society} OR {inclusive societies} OR {responsive institution} OR {responsive institutions} OR {fair society} OR {fair societies} OR {legal remedy} OR {legal remedies} OR {independence of judiciary} OR {independent judiciary} OR {separation of powers} OR extremism OR {war crime} OR {peaceful society} OR {organized crime} OR {illicit transfer} OR {illicit money} OR {arms trafficking} OR {cybercrime} OR {insurgence} OR {democratic institution} OR {political instability} OR ( {political decision-making} AND ( responsive OR inclusive OR participatory OR representative ) ) OR {Aarhus Convention} OR {press freedom} OR {freedom of speech} ) AND NOT ( {disease} OR {genetics} ) ) AND KEY(dental caries) | 148 |

Filters: source type= journal; keyword= dental caries, child, child preschool, preschool child

**WOS search strategy**

| # | Search Query | Results |
| --- | --- | --- |
| 1 | TS= actual innocence | 103 |
| 2 | TS= false confession | 714 |
| 3 | TS= armed conflict | 15557 |
| 4 | TS= civil conflict | 14799 |
| 5 | TS= war | 369291 |
| 6 | TS= warfare | 28595 |
| 7 | TS= democracy | 133616 |
| 8 | TS= Geneva Convention | 872 |
| 9 | TS= peace* | 87372 |
| 10 | TS= peacekeeping | 3549 |
| 11 | TS= corruption | 37658 |
| 12 | TS= government | 551907 |
| 13 | TS= bribery | 2737 |
| 14 | TS= crime | 120978 |
| 15 | TS= criminal | 99801 |
| 16 | TS= democrati* | 117427 |
| 17 | TS= politics | 389788 |
| 18 | TS= exoneration | 431 |
| 19 | TS= genocid* | 11133 |
| 20 | TS= homicid* | 16675 |
| 21 | TS= murder* | 22337 |
| 22 | TS= "human trafficking" | 3062 |
| 23 | TS= "justice system" | 12868 |
| 24 | TS= refugee* | 46273 |
| 25 | TS= refugee* | 46273 |
| 26 | TS= violence | 200185 |
| 27 | TS= torture | 8676 |
| 28 | TS= "arms flow" | 6 |
| 29 | TS= "transparent institution" | 5 |
| 30 | TS= "good governance" | 5807 |
| 31 | TS= "human rights" | 71234 |
| 32 | TS= "fundamental freedom" | 56 |
| 33 | TS= "child abuse" | 17429 |
| 34 | TS= "arbitrary detention" | 74 |
| 35 | TS= "organized crime" | 3847 |
| 36 | TS= "arms trafficking" | 67 |
| 37 | TS= insurgence | 704 |
| 38 | TS= "political instability" | 3183 |
| 39 | TS= "Aarhus Convention" | 212 |
| 40 | TS= "press freedom" | 910 |
| 41 | TS= "freedom of speech" | 2185 |
| 42 | #41 OR #40 OR #39 OR #38 OR #37 OR #36 OR #35 OR #34 OR #33 OR #32 OR #31 OR #30 OR #29 OR #28 OR #27 OR #26 OR #25 OR #24 OR #23 OR #22 OR #21 OR #20 OR #19 OR #18 OR #17 OR #16 OR #15 OR #14 OR #13 OR #12 OR #11 OR #10 OR #9 OR #8 OR #7 OR #6 OR #5 OR #4 OR #3 OR #2 OR #1 | 1936466 |
| 43 | TS= "dental caries" | 23146 |
| 44 | #43 AND #42 | 410 |

Filter: document type= article or review article or editorial material or proceeding paper

**URL to the figure if it needs editing**

https://viewer.diagrams.net/?tags=%7B%7D&highlight=0000ff&edit=_blank&layers=1&nav=1&title=flow%20chart3.png#R5Zpdb9owFIZ%2FDdJ2sSofTkouN6DdpHWqyrRuvTOJm1hyYuY4Bfbrd0wcQmJgHaUE0aqK4mPHH897bB%2B77bmDdH4t8DS54RFhPceK5j132HOcwEPwVIZFafD6l6UhFjQqTXZtGNM%2FRBstbS1oRPJGQck5k3TaNIY8y0goGzYsBJ81iz1y1mx1imNiGMYhZqb1nkYyKa1957K2fyY0TqqWbT8oc1JcFdYjyRMc8dmayR313IHgXJZv6XxAmGJXcSm%2Fu9qSu%2BqYIJl8zgfxzd384WY2C6PfXydCPAx49OODruUJs0IPeBzyaZH3HJ9BrZ8mAt5i9ZYtsy0b9fVw5KJiJHiRRUQ1Y0HBWUIlGU9xqHJn4BRgS2TKIGXDq9ntqg9ESDJfM%2BlhXBOeEikWUETn9jVR7VJIJ2e1PnYFPVnTxtc2rF0iXlVcU4MXDe4%2FIDoGxNtikgKQbRAdFHQO8eQougbFezIBA3%2BExzikJAMY24giy%2BucKDo1osggegf1Evq0wzX9ahAn5Jp25yQ9g%2BR3KhnsS6oXPk4Vg%2FKpWp%2FkUuBQqtw8FIRku3j73a%2Bnbd7de65v8L4qll%2FBiJZccZ4T%2BN3O1bb8k%2BPqds710uD6JQtZEe0AibpfWI2tqnOOfYPjaA4cc8ozML%2Br5rb9frU6ZJO8XB7amL9B%2FAetLWWgWaxCWSyoWloGqucQncIWyCIilPcnWNXvl3kZVwFtPS9U8SdMGZ6AULpEWTc8RlnMaJ6Y%2Fel60%2FSd5hwJNmjrH1Pb4Fnaer7zfpOYqkxSpEqnXfpZC4JF3lRpsweUuYIwLNUshdOPCoeG13b36xvy7ea%2B0bl21alxTbxhMWU0BHj5EmPKd0U%2FttM91HbwswnqURc72zwkGpRIFn1Up21IhQw2Zxo2wTQpAh2x%2BKkSF16V%2FLWeN5w3Ugud2opXYhET%2Be9QmESN074pwhpkbwPjyracjhBHNzqxCbxu4ZbTTNYarwLaSmOrpV3OCxES%2FdX6mb5dUdtZvFZFJRijoqUfrIb9Atcwj74vdI2tEp%2BIdJ53IOnctg%2BgI0tnnrfPXDrXDS76%2FpmoZ57t34R61uqnFXpY9mGEdJB9XCHNq4W3LWQQHEZIaOa4Qpp3Fm9aSHSopRUde2k1L0leKGQd7jaC3Tr2PXi4W52DTsVTUGuSO3v6BmqvFu2KXts3zIufc5%2FkLelce8%2F1GfnW7opeWzrzXufspXOaC%2Bnes651z2NUtLd0kKz%2FAF4Wr%2F%2BLwB39BQ%3D%3D
